# Improving performance in medical practices through the extended use of electronic medical record systems: a survey of Canadian family physicians

**DOI:** 10.1186/s12911-015-0152-8

**Published:** 2015-04-14

**Authors:** Louis Raymond, Guy Paré, Ana Ortiz de Guinea, Placide Poba-Nzaou, Marie-Claude Trudel, Josianne Marsan, Thomas Micheneau

**Affiliations:** 1 Université du Québec à Trois-Rivières, Trois-Rivières, Canada; 2 Chair in Information Technology in Health Care, HEC Montréal, 3000, Chemin de la Côte-Sainte-Catherine, Montréal, Québec H3T 2A7 Canada; 3 Université du Québec à Montréal, Montréal, Canada; 4 Université Laval, Québec City, Canada

**Keywords:** Electronic medical records, Primary care, Family physicians, Extended use, User satisfaction, Ease of use, Functional coverage, Survey research, Structural equation modeling

## Abstract

**Background:**

Numerous calls have been made for greater assimilation of information technology in healthcare organizations in general, and in primary care settings in particular. Considering the levels of IT investment and adoption in primary care medical practices, a deeper understanding is needed of the factors leading to greater performance outcomes from EMR systems in primary care. To address this issue, we developed and tested a research model centered on the concept of Extended EMR Use.

**Methods:**

An online survey was conducted of 331 family physicians in Canadian private medical practices to empirically test seven research hypotheses using a component-based structural equation modeling approach.

**Results:**

Five hypotheses were partially or fully supported by our data. Family physicians in our sample used 67% of the clinical and 41% of the communicational functionalities available in their EMR systems, compared to 90% of the administrative features. As expected, extended use was associated with significant improvements in perceived performance benefits. Interestingly, the benefits derived from system use were mainly tied to the clinical support provided by an EMR system. The extent to which physicians were using their EMR systems was influenced by two system design characteristics: functional coverage and ease of use. The more functionalities that are available in an EMR system and the easier they are to use, the greater the potential for exploration, assimilation and appropriation by family physicians.

**Conclusions:**

Our study has contributed to the extant literature by proposing a new concept: Extended EMR Use. In terms of its practical implications, our study reveals that family physicians must use as many of the capabilities supported by their EMR system as possible, especially those which support clinical tasks, if they are to maximize its performance benefits. To ensure extended use of their software, vendors must develop EMR systems that satisfy two important design characteristics: functional coverage and system ease of use.

## Background

### The need for the study

In the face of rapidly increasing healthcare costs, associated with an aging population and the concomitant rise in chronic illnesses [1], governments in developed countries such as the United Kingdom and Canada have felt obliged to improve the efficiency and effectiveness with which primary medical care is provided to their citizens. This has led, for instance, to a greater emphasis on the prevention and monitoring roles played by family physicians and other healthcare professionals [2]. Another important effect has been the deployment of information technology (IT) in support of the incremental and radical changes made by these governments to their healthcare systems. Of all the IT-based applications currently in use in primary care settings, electronic medical records (EMR) have the most wide-ranging capabilities [3] and thus the greatest potential for enhancing health care services. An EMR can be defined as a computerized system where physicians record relevant information such as patient demographics, medical histories, consultation notes, lists of problems, allergies, vaccinations, vital signs, and prescriptions/renewals [4]. Some EMR systems may also contain other functionalities such as automated alerts, medical appointments and reminders. In short, an EMR system is designed to support the needs of individual physicians who are directly caring for patients in their medical practices^a^.

A recent survey conducted by the Commonwealth Funds revealed high adoption rates of EMRs by family physicians in New Zealand, Australia and several European countries, including the Netherlands, the United Kingdom, Sweden and Germany [5]. So the potential value of these systems seems to be widely recognized, yet prior research shows that significant challenges remain before such benefits can be reaped. Recent systematic reviews on this topic found either inconclusive or mixed effects of EMR systems on performance outcomes. For instance, Lau et al. [6] concluded that, based on prior empirical studies, there is a 51% chance that an EMR system will improve office practices, a 30% likelihood that it will have no effect, and a 19% chance that it will lead to negative outcomes. Similarly, Holroyd-Leduc et al. [7] found that while EMR systems appear to have clear advantages over traditional paper-based records in terms of legibility and accessibility, effects on work processes (e.g., quality of care, individual efficiency and productivity) and clinical outcomes (e.g., blood pressure control, glycemic control) have not yet been found.

Previous research reveals that the lack of perceived benefits from EMRs may be at least partially attributed to underutilization of these systems by physicians, which may be related to users’ attitudes toward EMRs. For instance, Miller and Sim [3], who conducted interviews with several EMR users, observed that attaining benefits depends heavily on physicians’ use of EMR functionalities. More recently, Price et al.’s [8] qualitative analysis showed there are “ceiling effects” to EMR use in primary care practices owing to numerous factors, including a lack of awareness or availability of EMR functionalities to support clinical work as well as the poor usability of these systems. Similarly, Bouamrane and Mair [9], who also carried out in-depth interviews with general practitioners, concluded that there remains substantial scope for improving family physicians’ interactions and overall satisfaction with these systems.

In light of these findings, the present study examines how EMR systems are actually being used by family physicians, which system design characteristics influence physicians’ usage patterns, and the extent to which EMR use is associated (or not) with particular performance benefits. More specifically, we sought answers to the following research questions: 1) How can the use of EMR systems by family physicians in medical practices best be characterized? 2) To what extent does the use of an EMR system by family physicians, as well as their satisfaction with the system’s functionalities, influence performance benefits? and 3) Do the design characteristics of a specific EMR system determine the extent to which family physicians use the system and are satisfied with it?

### Research model and hypotheses

**Figure 1:**
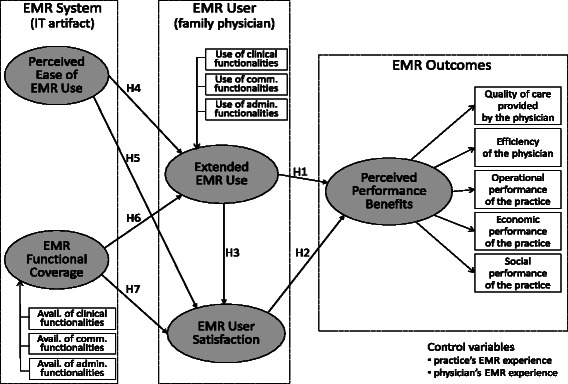
Research Model.

Prior EMR acceptance research has mainly focused on the basic dichotomous adoption decision (yes/no) or amount of system usage (i.e. frequency, time and extent) (e.g., [10,11]). We posit that such limited theoretical attention to EMR usage may explain our lack of knowledge about its impacts on performance outcomes in primary care settings. To overcome such limitations, our research model (see Figure 1) is built around the concept of “Extended Use,” which we have borrowed from the field of information systems (IS).

Saga and Zmud [12] first coined the term “extended use”, broadly describing it as individuals using more of the technology’s features in order to carry out a more comprehensive set of work tasks. More recently, Hsieh and Wang [13] defined extended system usage as “the use behaviour that goes beyond typical usage and can potentially lead to better results and returns” (p.217). Prior research in the medical informatics field has shown that the use of systems such as PACS (picture archiving and communication system), clinical decision support systems and CPOE (computerized physician order entry) can have positive effects on performance outcomes (e.g., [14-16]). However, the main implication of these findings is that such benefits are not usually associated with the mere adoption of these systems, but rather with their extensive use [3,17]. Hence, the first research hypothesis is formulated as follows:

*Hypothesis 1 – Extended use of an EMR system is positively and significantly associated with performance benefits.*

One of the premises of this study is that the value created through system usage must be measured from the user’s perspective [18]. In this regard, the user’s attitude toward a system, defined as “user satisfaction”, has been found to predict performance or beneficial impacts better than his or her usage behaviours [19]. The argument here is that while user satisfaction may not “cause” performance *per se*, it indicates that the new system has met individual and organizational expectations [20] and, in turn, that the performance improvements expected from its use should ensue [21]. Furthermore, previous studies of inpatient clinical information systems have also confirmed that user satisfaction is a pre-requisite to the attainment of performance benefits (e.g., [22,23]). Hence, our second hypothesis is formulated as follows:

*Hypothesis 2 - EMR user satisfaction is positively and significantly associated with performance benefits.*

Prior empirical findings demonstrate that user satisfaction increases as training, experiential learning or improved facilitating conditions renders IS use more effective over time (e.g., [24]). The empirical evidence supporting a direct influence of system usage on user satisfaction, however, has been rather weak [25]. Still, a more frequent and intensive use of clinical information systems has been previously associated with increased user satisfaction. For instance, a recent study by Maillet et al. [26] reveals that the extent to which registered nurses use an EHR (electronic health record) system to support their work in acute care settings influences their level of satisfaction with such a system. Hence, our third hypothesis is formulated as follows:

*Hypothesis 3 – Extended use of an EMR system is positively and significantly associated with user satisfaction.*

Prior research clearly indicates that user behaviours and attitudes toward a system are predicated upon the system’s ease of use, i.e. the extent to which users perceive system use to be free of effort [27-31]. For one thing, the system’s ease of use initially facilitates exploratory usage behaviours on the part of users, leading them to experiment with and eventually use a wider range of the system’s functionalities [32]. Furthermore, previous research has also found a system’s ease of use positively influences user satisfaction. For instance, a study by Jaspers et al. [33] found that physicians’ satisfaction with a redesigned EMR system was closely related to their perceptions not only of its enhanced functionality but also of its improved usability. In line with these studies, our next two hypotheses can be formulated as follows:

*Hypothesis 4 - An EMR system’s ease of use is positively and significantly associated with extended use of the system.*

*Hypothesis 5 - An EMR system’s ease of use is positively and significantly associated with user satisfaction.*

Users’ behaviours and attitudes are also predicated upon the system’s functional coverage, i.e. the extent to which the system “includes the features that the organization needs to operate and that users need to do their work” [34:746]. It stands to reason that the greater the number of functionalities available in a system, the greater the potential for their exploration, assimilation and appropriation by physicians [34,35]. For example, Sicotte et al. [36] found that physicians were more satisfied with a PACS system when they perceived it to be more useful, i.e. when it provided “a complete range of functionalities” in support of their work, was “well harmonized” with their clinical practices, and was “compatible” with all aspects of their tasks. These findings lead to the formulation of the following hypotheses:

*Hypothesis 6 - Functional coverage of an EMR system is positively and significantly associated with its extended use.*

*Hypothesis 7 - Functional coverage of an EMR system is positively and significantly associated with user satisfaction.*

As shown in Figure 1, our research model includes two control variables: individual and organizational experience with EMRs. We posit that individual experience may influence physicians’ behaviours and attitudes toward EMR systems through a process of experiential “learning by doing,” [37] while organizational experience may exercise such influence through a process of infusion or institutionalization [38].

## Methods

An online survey was conducted of family physicians in private medical practices in the province of Québec, Canada to empirically test the research model. The questionnaire instrument, which was developed in both French and English, was initially validated by representatives of the study’s sponsors, Canada Health Infoway and the Quebec Federation of General Practitioners (QFGP).

### Data collection and sample

An invitation to participate in the study was emailed to 4,845 members of the QFGP who were practising in primary care settings and could be reached by email. The invitation letter, co-signed by a high-ranking officer of the QFGP, contained a hyperlink directing the family physicians to the questionnaire through a secure Web site. The online questionnaire was developed on the Qualtrics online survey platform [39]. Seven days after the first invitation, a reminder letter was emailed to all members of the survey’s target population. Ethical approvals were obtained from HEC Montréal in April 2013.

Data was thus collected from 780 family physicians, representing a 16% response rate. Low response rates raise the possibility of non-response bias. As suggested by Hikmet and Chen [40], the potential for such a bias was first assessed by comparing the 156 “late” respondents (i.e. those responding after having received the reminder) with the 624 “early” respondents. Our analyses indicate no statistically significant differences between these two sets of respondents. While this must be interpreted as an encouraging sign, researchers must remain vigilant about response rates if they are uncertain about sample representativeness [41], so as a second step we compared the demographic characteristics of our respondents with those of the target population, i.e. QFGP members. Our sample was found to be statistically representative of the population in terms of physician age, gender, region and length of professional experience. In short, these two assessments indicate that sampling error is unlikely, and thus give confidence that our sample approximates the characteristics of the target population [42].

Whereas the survey initially targeted two different types of family physicians – those working in primary care practices with or without an EMR system – this article only analyzes responses from the 331 family physicians who were actually using such a system. The results from the survey of doctors working in medical clinics without an EMR can be found elsewhere [43]. Of the 331 EMR users, 48% were women. As for age, 38% of the users were in their 50s, 28% in their 40s, and 19% in their 30s. Respondents had 22 years’ experience in the medical profession on average, with a minimum of 2 years and a maximum of 45 years. They also had an average of 4 years’ experience using their medical practice’s EMR system, and 56% of the sampled physicians had 3 years’ experience or less.

### Measurement

In order to measure the functional coverage of an EMR system and the extent of its use based on a list of twenty-four EMR functionalities (see Table 1), respondents were asked to indicate whether each functionality was available or not in their system, and if it was available, whether or not they actually used it. They were also asked to indicate their level of satisfaction with each of these functionalities, on a scale of 1 (very dissatisfied) to 5 (very satisfied). The list of functionalities was developed from previous empirical studies on the actual use of EMR systems in primary care settings [10,44-48].

**Table 1:** **EMR system functionalities available to and used by family physicians**

EMR system functionalities	Functional category	Functionalities available	Functionalities used
	Functionality	(% of systems)	(% of physicians)
**Clinical functionalities**	**Clinical Notes and Patient History**		
	Clinical notes	89%	83%
	Past medical history	90%	79%
	Family diseases/family history	86%	75%
	**Patient Care Management**		
	Planning and coordination of patient care	64%	55%
	Monitoring patients with chronic diseases	68%	53%
	Reminder for guideline-based interventions and/or screening tests	43%	29%
	**Prescription Management and Patient Demographics**		
	Patient demographics	89%	82%
	Electronic alerts or prompts about a potential problem with drug use, dose or drug interactions	79%	59%
	Electronic prescribing of medication	89%	80%
**Communication functionalities**	**Visualization of Results**		
	All lab tests ordered are tracked until results reach clinicians	46%	39%
	Viewing laboratory test results	88%	82%
	Out-of-range test levels highlighted	65%	60%
	Viewing imaging results	54%	51%
	**Communication with other Institutions**		
	Access to medical records from other clinics/hospitals	36%	34%
	Communication and follow-up with government agencies (e.g. a public health agency)	22%	18%
	Electronic ordering of laboratory tests	49%	34%
	Electronic referral to specialists	31%	27%
	**Electronic Transfers**		
	Electronic transfer of prescription to pharmacy	15%	10%
	Electronic transfer of test prescription to laboratory	44%	39%
**Administrative functionalities**	**Billing and Data Security**		
	Billing management	78%	42%
	Secure transmissions protecting patients’ health privacy	69%	61%
	**Remote Access and Appointment Scheduling**		
	Accessibility of EMR remotely (from home or outside the clinic)	89%	68%
	Accessibility of EMR when traveling (train, airport, mobile)	66%	35%
	Patient appointment schedule management	92%	79%

A principal components exploratory factor analysis (EFA) was first performed to empirically – rather than theoretically – group the EMR functionalities used by the surveyed family physicians into eight functional categories. As shown in Table 1, each of these categories was in turn determined to be part of an EMR system’s clinical, communicational or administrative capabilities. This grouping of EMR functionalities is based on descriptions in the literature of activities performed by primary care physicians – such as prescribing medication, ordering laboratory tests or billing patients – as being either part of a clinical process (e.g., [49]), a communication process (e.g.,[50]) or an administrative process (e.g., [51]).

Using a set of 24 potential outcomes of EMR system usage (see Table 2), also extracted from the extant literature [52-65], respondents were asked to what extent they agreed (where 1 = strongly disagree and 5 = strongly agree) with statements about the impacts of EMR usage on their individual performance (e.g. “has facilitated my application of clinical care guidelines for my patients”) and on their clinic’s performance (e.g. “has improved teamwork and the continuity of care provided to patients”). A confirmatory factor analysis (CFA) was performed to ascertain the reliability and validity of the perceived performance benefits construct. As presented in Table 2, the assumed penta-dimensional structure of this construct was confirmed, as well as the five measures’ composite reliability and convergent validity.

**Table 2:** **Confirmatory factor analysis of Perceived performance benefits**

Perceived Performance Benefits	Loadings				
	F1	F2	F3	F4	F5
**Individual performance improvements (family physician)**					
Improved the quality of preventive care given to my patients	.86	-	-	-	-
Improved the monitoring of my patients with chronic diseases	.82	-	-	-	-
Improved the safety of care for my patients	.69	-	-	-	-
Facilitated application of clinical care guidelines for my patients	.85	-	-	-	-
Facilitated my clinical decision making	.81	-	-	-	-
Improved the quality of prescriptions to my patients	.61	-	-	-	-
Improved the quality of my documentation and clinical notes	-	.70	-	-	-
Improved my communications with health care providers	-	.74	-	-	-
Allowed me to use clinical resources more wisely	-	.78	-	-	-
Reduced the average length of my patient consultations	-	.78	-	-	-
Allowed me to be more efficient	-	.79	-	-	-
Improved communications and interactions with my patients	-	.80	-	-	-
Reduced my time spent on medical documentation and ordering	-	.71	-	-	-
**Organizational performance improvements (family practice)**					
Improved the efficiency of the clinic’s staff	-	-	.60	-	-
Improved teamwork and the continuity of care	-	-	.62	-	-
Decreased the clinic’s operating costs	-	-	-	.70	-
Reduced the number of “no shows”	-	-	.72	-	-
Increased the clinic’s revenues	-	-	-	.83	-
Improved the quality of services delivered to the clinic’s patients	-	-	.82	-	-
Decreased the number of patient visits to the clinic	-	-	.76	-	-
Improved collaboration with other clinical care providers	-	-	.73	-	-
Improved prescription management at the clinic	-	-	.64	-	-
Increased access to care for the community served by the clinic	-	-	-	-	.89
Increased immunization rates in the community we serve	-	-	-	-	.77
**Composite reliability**	.90	.90	.87	.74	.82
**Average variance extracted**	.61	.58	.49	.59	.69

^a^On a 5-point Likert scale (1: strongly disagree, 2: disagree, 3: neutral, 4: agree, 5: strongly agree).

F1: Quality of Care provided by the physician.

F2: Efficiency of the physician.

F3: Practice’s workflow (Operational Performance).

F4: Practice’s financial position (Economic Performance).

F5: Practice’s community (Social Performance).

Similarly, a CFA of the EMR User Satisfaction construct was conducted to verify the tri-dimensional structure assumed, i.e. satisfaction with the EMR system’s clinical, communicational, and administrative functionalities. The results of this analysis, presented in Table 3, also confirm this to be true, as well as the three measures’ composite reliability and convergent validity.

**Table 3:** **Confirmatory factor analysis of EMR User Satisfaction**

EMR User satisfaction ^ a ^	Loadings		
	F1	F2	F3
	Clinical Functionalities	Communication Functionalities	Administrative Functionalities
*with EMR system functionalities*			
Clinical notes and patient history	.66	-	-
Patient Care Management	.75	-	-
Prescription management and patient demographics	.83	-	-
Visualization of results	-	.74	-
Communications with other institutions	-	.78	-
Electronic transfers	-	.80	-
Billing and data security	-	-	.69
Remote access and appointment scheduling	-	-	.72
**Composite reliability**	.79	.82	.66
**Average variance extracted**	.56	.60	.50

^a^On a 5-point scale (1: very dissatisfied, 2: dissatisfied, 3: neutral, 4: satisfied, 5: very satisfied).

The instrument measuring the Perceived Ease of EMR Use construct was taken from the Commonwealth Fund’s international survey of family physicians [5]. It is composed of eight 5-point scales assessing the ease with which a physician can use an EMR system to gather information for clinical reporting and decision making purposes (1: unable to generate this information, 2: difficult, 3: neutral, 4: easy, 5: very easy to generate this information). As shown in Table 4, an EFA of this construct produced a bi-factorial structure, with one measure of ease of use with regard to patients and another with regard to care providers.

**Table 4:** **Exploratory factor analysis of the Perceived Ease of EMR Use**

Perceived Ease of EMR Use ^ a ^	Loadings	
	F1	F2
	with regard other care providers	with regard patients
List of patients by diagnosis (e.g. diabetes, cancer)	.85	-
List of patients by specific laboratory result (e.g. HbA1C > 0.9)	.79	-
List of patients who are due or overdue for tests or preventive care (e.g., flu vaccine due)	.78	-
List of all medications taken by an individual patient (including those that may be prescribed by other doctors)	-	.82
List of all patients taking a particular medication	.80	-
List of all laboratory results for an individual patient (including those ordered by other doctors)	-	.83
List of patients registered in the doctor’s name	.64	-
List of patients vulnerable or not vulnerable to specific diagnoses/disease cohorts	.71	-
**Eigen value**	3.7	1.7
**Explained variance**	41%	19%

^a^On a 5-point scale (1: unable to generate this information, 2: difficult, 3: neutral, 4: easy, 5: very easy).

Finally, our respondents were asked questions aimed at contextualizing their use of EMRs in terms of their individual and medical practice demographics, including the physician’s and the practice’s experience with EMR systems, which represent the research model’s two control variables.

## Results and discussion

As shown in Figure 1, one exogenous construct in the research model, EMR Functional Coverage, and one endogenous construct, Extended EMR Use, have been modeled as being “formative” rather than “reflective”, given their composite and multidimensional nature. Since each formative indicator captures a different aspect of the model, changes in these indicators bring about or “cause” change in their underlying construct [66]. Component-based structural equation modeling (SEM) was used to validate our research model; Partial Least Squares (PLS) was employed because it is better suited to measurement models that include both exogenous and endogenous formative constructs [67].^b^ As implemented in SmartPLS software [68], this approach was also chosen for its robustness with regard to the distribution of residuals [69]^c^.

### Test of the measurement model

Table 5 presents descriptive statistics, reliability coefficients and the correlation matrix of the research variables. The first step in the data analysis consisted of simultaneously estimating the measurement and structural models using PLS. Psychometric properties of both the formative and reflective construct indicators (measures) were thus assessed within the context of our research model.

**Table 5:** **Descriptive statistics, reliability and inter-correlation of the research variables**

Variable (range)	mean	s.d.	α ^ a ^	VIF ^ b ^	1.	2.	3.	4.	5.	6.	7.	8.	9.	10.	11.	12.	13.	14.	15.	16.	17.
Performance Benefits (1–5)																					
1. Quality of Care	2.9	1.0	.91	-	-																
2. Efficiency of physician	2.9	1.0	.91	-	.82	-															
3. Operational Performance	3.0	0.9	.90	-	.77	.84	-														
4. Economic Performance	2.7	1.0	.73	-	.58	.68	.74	-													
5. Social Performance	2.5	1.0	.82	-	.71	.76	.79	.64	-												
Extended EMR Use (0–1)^c^																					
6. Use of Clinical functionalities	.58	.24	-	1.39	.42	.41	.40	.29	.34	-											
7. Use of Communication funct.	.34	.26	-	1.38	.29	.30	.28	.23	.20	.51	-										
8. Use of Administrative funct.	.81	.30	-	1.11	.19	.21	.21	.07	.08	.28	.27	-									
EMR User Satisfaction (1–5)																					
9. With Clinical functionalities	3.7	1.0	.93	-	.39	.39	.40	.25	.32	.14	.13	.12	-								
10. With Communication funct.	3.8	1.1	.89	-	.29	.32	.33	.22	.24	.01	-.02	.13	.51	-							
11. With Administrative funct.	4.2	0.9	.77	-	.23	.26	.27	.17	.20	.19	.16	.06	.65	.43	-						
Perceived Ease of EMR Use (1–5)																					
12. With regard to Patients	2.8	1.2	.61	-	.41	.40	.42	.34	.27	.34	.23	.13	.23	.20	.24	-					
13. With regard to Care providers	1.9	0.9	.85	-	.40	.38	.35	.29	.33	.23	.20	.09	.19	.08	.11	.37	-				
EMR Functional Coverage (0–1)^d^																					
14. Availability of Clinical funct.	.67	.22	-	1.39	.36	.35	.34	.23	.30	.83	.43	.24	.11	.01	.12	.25	.23	-			
15. Availability of Comm. funct.	.45	.25	-	1.34	.26	.25	.24	.20	.20	.43	.80	.22	.17	-.03	.14	.19	.18	.50	-		
16. Availability of Admin. funct.	.90	.22	-	1.08	.17	.14	.19	.04	.10	.23	.15	.69	.10	.08	.08	.13	.13	.27	.18	-	
Control variables																					
17. Physician’s EMR experience (y.)	4.1	3.7	1.0	-	.26	.27	.25	.23	.14	.03	.09	.13	.21	.18	.17	.15	.18	-.02	.02	.04	-
18. Practice’s EMR experience (y.)	15.2	4.1	1.0	-	-.27	-.27	-.25	-.23	-.18	-.10	-.17	-.16	-.22	-.10	-.21	-.13	-.16	-.04	-.09	-.09	-.83

^a^Cronbach’s reliability coefficient [inappropriate for index variables].

^b^Variance inflation factor [formative indicators only].

^c^No. of functionalities used/total no. of functionalities.

^d^No. of functionalities available/total no. of functionalities.

*Nota.* Correlations greater than 0.11 are significant (two-tailed, p < 0.05).

Given that the usual reliability and validity criteria do not apply to a formative construct, it must first be verified that there is no multicollinearity among the indicators forming this construct [70]. This was verified with the variance inflation factor (VIF), based on the guideline that this statistic should not be greater than 3.3 for any formative indicator [71].^d^ As shown in Table 5, this condition held for all six indicators.

**Figure 2:**
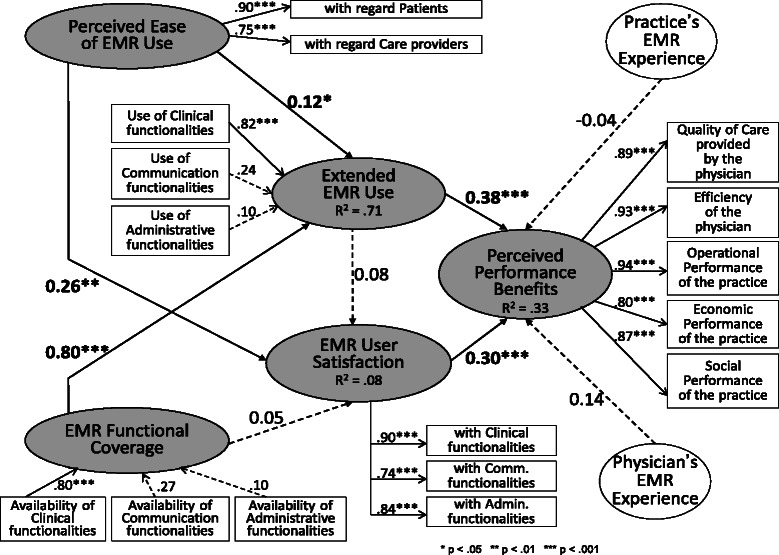
Test of the Research Model (PLS, n = 331).

Once the validity of the formative constructs has been assessed, the unidimensionality and reliability of the reflective constructs must then be evaluated. As depicted in Figure 2, the fact that all indicator loadings (λ) on these constructs were greater than the 0.7 threshold confirmed their unidimensionality. As shown in Table 6, composite reliability coefficient values above the 0.7 threshold also provide strong evidence of the reliability of the three reflective constructs. Evidence of the convergent validity of the reflective constructs was also found, as their average variance extracted (AVE) values are all above the 0.5 threshold.

**Table 6:** **Reliability, convergent and discriminant validity of the research constructs**

Construct	c.r. ^ a ^	1.	2.	3.	4	5.	6.	7.
1. Perceived Ease of EMR Use	.81	.82^b^						
2. EMR Functional Coverage	-	.31^c^	-					
3. Extended EMR Use	-	.37	.83	-				
4. EMR User Satisfaction	.87	.27	.13	.16	.83			
5. Perceived Performance Benefits	.95	.49	.38	.40	.40	.89		
6. Practice’s EMR Experience	1.0	-.17	-.06	.06	-.22	.26	1.0	
7. Physician’s EMR experience	1.0	.19	-.01	-.13	.22	-.27	-.81	1.0

^a^Composite reliability coefficient = (Σλ_i_)^2^/((Σλ_i_)^2^ + Σ(1-λ_i_^2^)) [inappropriate for formative constructs].

^b^Diagonal: (average variance extracted)^1/2^ = (Σλ_i_^2^/n)^1/2^ [inappropriate for formative constructs].

^c^Subdiagonals: correlation = (shared variance)^1/2^.

The last property to be verified is discriminant validity, which shows the extent to which each construct in the research model is unique and different from the others. Here, the shared variance between a reflective construct and other constructs must be less than the AVE by a construct from its indicators. The results in Table 6 show this to be the case for all three reflective constructs included in the model. For the two formative constructs, discriminant validity is demonstrated by a correlation with any other construct that is significantly different from unity (at p < 0.001) [72].

### Test of the structural model

The research hypotheses were tested by assessing the path coefficients (β) estimated by PLS (see Figure 2). While PLS does not provide model fit indices, the performance of the structural model can be ascertained by the strength and significance of the path coefficients and by the proportion of construct variance (R^2^) that it explains [69]. Moreover, only path coefficients greater than 0.2 should be considers “truly” significant, since PLS tends to underestimate structural paths when compared with covariance structure-based approaches, such as EQS or LISREL [73].

#### Hypothesis 1 (confirmed)

As shown in Figure 2, a positive and significant path coefficient (ß = 0.38, p < 0.001) confirms the hypothesis that extended use of an EMR system by family physicians leads to improvements in performance benefits. Further examining the weights of the three indicators that “form” the “extended EMR use” construct, one finds that the “use of clinical functionalities” indicator is by far the most determinant (γ = 0.82, p < 0.001). The concept of extended use and the performance benefits to be obtained from such use are thus mainly tied to the clinical support provided by an EMR system through patient care-oriented functionalities such as e-prescriptions and the monitoring of chronically-ill patients.

#### Hypothesis 2 (confirmed)

The hypothesis that increasing physicians’ satisfaction with an EMR system leads to increased performance benefits for the physicians and their practices is affirmed by a positive and significant path coefficient (ß = 0.30, p < 0.001). This implies that governments and medical associations should not only encourage more extended use of EMRs by family physicians, but that they should also encourage EMR software designers and vendors to offer systems whose clinical, communicational and administrative functionalities meet user requirements *in their contexts* and, thus, better satisfy physicians. Since these requirements vary by physician and by medical practice, the preceding results allow one to associate specific user requirements with specific individual and organizational performance benefits, thus providing an empirical basis for greater flexibility and customization of EMR systems, whether through proprietary or open-source software [74].

#### Hypothesis 3 (unconfirmed)

Given a non-significant path coefficient (ß = 0.08, p > 0.05), the hypothesis that more extended use of EMRs by family physicians increases user satisfaction could not be confirmed. As mentioned above in the formulation of the hypothesis, prior empirical results in this regard have been mixed. Here, the intended consequences of EMR usage (performance improvements) are seen to have both a behavioural determinant (extended use) and an attitudinal determinant (user satisfaction), and these determinants act independently.

#### Hypothesis 4 (partly confirmed)

Returning to Figure 2, a positive and significant – though not very strong – path coefficient (ß = 0.12, p < 0.05) partially confirms the hypothesis that physicians use EMRs more extensively when they perceive such usage to be free of effort. This result is aligned with the findings of Price et al. [8] that family physicians tend not to use those EMR features that they find difficult to use or disruptive to patient care work flow. In further analyzing this relationship, one finds that it is strongest between the system’s ease of use with regard to patients and the physician’s use of clinical functionalities (see the correlations in Table 5). This latter result again emphasizes the patient care- and clinical process-centered view of the extended use of EMRs.

#### Hypothesis 5 (confirmed)

The positive and significant path coefficient (ß = 0.26, p < 0.01) also confirms the hypothesized relationship between an EMR system’s ease of use and physicians’ satisfaction with using it. While this is hardly surprising given previously-cited IT user satisfaction surveys, this result nonetheless adds validity and generalizability to the nomological network linking the five research constructs in our research model.

#### Hypothesis 6 (confirmed)

The hypothesis that a wider functional coverage of an EMR system leads physicians to use the system in a more extensive way is fully confirmed, as the corresponding path coefficient is shown to be strongly positive and significant (ß = 0.80, p < 0.001). Examining the weights of the three formative indicators of the “EMR functional coverage” construct, one finds the “availability of clinical functionalities” indicator to be by far the most determinant (γ = 0.80, p < 0.001). Notwithstanding such a positive finding, much like Price et al. [8] we observed an important “ceiling effect” in terms of EMR usage by family physicians in private medical practices. Indeed, the results in Table 5 indicate that surveyed physicians only use, on average, 67% of the clinical functionalities and 47% of the communication functionalities in their EMRs. This compares with their use of 90% of its administrative features. A lack of awareness among physicians of EMR capabilities may explain, at least in part, this gap between what is available and what is actually being used (see Table 1). To minimize the “ceiling effect” problem, it is recommended that training and user-group sessions be organized on a regular basis (rather than only when the EMR system is first implemented). Such sessions or meetings would appear to be particularly important when new versions of an EMR product are released to the user community. Importantly, EMR vendors must carefully manage their clients’ expectations and ensure that clients have a clear understanding of the capabilities and limitations of their products, to avoid unnecessary dissatisfaction.

#### Hypothesis 7 (unconfirmed)

Given a non-significant path coefficient (ß = 0.05, p > 0.05), the last research hypothesis could not be confirmed. Thus, the functional coverage of an EMR system seems to have no bearing on physicians’ satisfaction with its various functionalities. In light of the previous finding that extended use also has no direct bearing on user satisfaction, one may surmise that family physicians generally have much higher expectations regarding the ease of use of an EMR system than its functional coverage.

Returning to Figure 2, the “extended EMR use” and “EMR user satisfaction” constructs were found to jointly explain 33% of the variance in the dependent variable. This represents a “strong” effect size, in that it is very close to the 35% threshold [75]. Lastly, no significant effect could be observed for the two control variables: the physician’s and the practice’s experience with EMRs.

### Test of the adjusted research model

**Figure 3:**
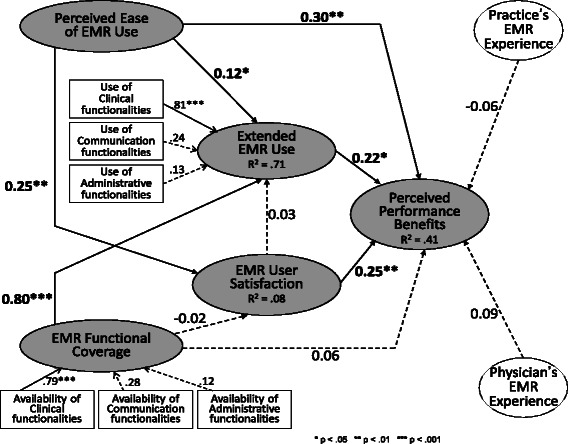
Test of the Adjusted Research Model (PLS, n = 331).

Further analysis was performed by making two adjustments to the initial model. First, in line with a position taken previously by certain IS researchers [13,76], the third hypothesis was reformulated by reversing the causal direction of the relationship posited between extended use and user satisfaction. Second, in line with previous IS studies [45,77], the EMR artefact’s characteristics (perceived ease of use and functional coverage) were posited to have direct effects on the attainment of performance benefits, i.e. irrespective of the extent to which physicians use the system and are satisfied with it. Figure 3 presents the test results from the adjusted structural model with PLS.

First, given a non-significant path coefficient (ß = 0.03, p > 0.05) estimated from the second PLS structural analysis, the following alternative to the third hypothesis could not be confirmed:

*Hypothesis 3*_*alt*_ *(unconfirmed) - EMR user satisfaction is positively and significantly associated with extended EMR use.*

In light of the previously-cited studies, this additional result provides even more reason to clearly distinguish EMR user satisfaction and extended EMR use in terms of their components and determinants, since the mutual dependence of these two constructs was found to be low.

Second, a positive and significant path coefficient (ß = 0.30, p < 0.01) confirms the following hypothesis:

*Hypothesis 8 (confirmed) - An EMR system’s ease of use is positively and significantly associated with performance benefits.*

The intended consequences of EMR adoption and assimilation in primary care settings are thus found to have an added artefactual determinant (the EMR system), i.e. irrespective of the behavioural (extended use) and attitudinal (user satisfaction) determinants previously demonstrated. This result again underscores the need to account for EMR design characteristics in explanations of the benefits to be obtained from EMR use.

As IT artefacts, prior research has often “blackboxed” EMR systems or “reduced them to surrogate measures” [78]. The preceding results provide an empirical basis for EMR design and evaluation that is coherent with the particular individual (family physician) and organizational (primary care clinic) contexts of EMR use. EMR designers and vendors may therefore find it useful to employ the empirical description of EMR systems provided in this study to pinpoint the functional features that most and least satisfy users, and whose usage is most or least conducive to performance. To this end, Tables 1, 2, 3 may provide an initial reference framework. These results may also guide the evaluation and improvement of existing systems, such as highlighting their missing features, since family physicians and other primary care professionals generally have no benchmarks for EMR functionality and effectiveness to support their decision making on these issues [79].

Overall, the adjusted model explained 41% of the variance in the performance benefits perceived by family physicians. This represents a significant increase from the initial model. The revised model thus performed well in its nomological integration of the EMR artefact, the primary care physicians who use this artefact extensively, and the performance outcomes of such use.

## Conclusions

By developing and empirically testing a research model around the concept of “Extended EMR Use,” this study has produced original and novel results that may eventually apply to other types of clinical information systems deployed in other settings. Initial findings include the determination of what actually constitutes extended use of an EMR system, and the confirmation that extensive use of an EMR, and especially its clinical functionalities, is positively and significantly associated with performance benefits. The results of this study also confirm that the design characteristics of an EMR system, in terms of functional coverage and ease of use, determine the extent to which family physicians will make extensive use of the system.

As mentioned above, previous studies on this topic have focused either on identifying the benefits ensuing from physicians’ adoption of this technology, or on determining their “intention to use” EMR systems. In answering the call for more assimilation of IT in healthcare organizations, our study concentrated instead on physicians’ actual usage behaviours and on the characteristics of the EMR systems available to them [80], as we contend that a deeper understanding of EMRs as they are actually being implemented and used in primary care settings is a prerequisite to fully explaining whether or not and why performance improvements are achieved. Moreover, previous studies have generally taken a “generic” or theoretical approach to characterizing the functionalities of EMR systems, and have focused on specific features, such as electronic communication, or on individual functionalities, such as the monitoring of chronically-ill patients. Our study complements previous research since we founded our conceptualization on the reality of physicians’ actual use of EMRs in primary care medical practices.

Lastly, our results must be considered in light of the study’s low response rate and the inherent limitations of survey research, as there may yet be biases linked to the perceptual nature of the data. In particular, measuring all variables through a self-administered questionnaire and using single respondents may pose a risk of common method bias (CMB) and lead the relationships between constructs to be overestimated, such that basic precautions must be taken to minimize this risk [66]. Thus, the questionnaire was designed to be anonymous, giving the respondents all the latitude needed to express their true perceptions, attitudes and behaviours. Robust measurement scales were used, and the independent and dependent variables were placed in different sections of the questionnaire. Different question formats and scale types were also used for the five sets of variables measuring the theoretical constructs. Moreover, given that the potential for CMB is very rarely acknowledged in medical informatics survey research, with a few exceptions [81,82] we used the recommended “latent method factor” approach to further examine this issue [83], allowing us to conclude that CMB is not a major threat in this study^e^.

### Endnotes

^a^While the concepts of EMR and EHR (electronic health record) are sometimes used interchangeably, like other authors we consider them to be two distinct health information systems. An EHR system is an “aggregated, longitudinal system of systems which assembles health information about a patient over a wide area network from, potentially, many geographically dispersed data sources” [4:24]. It usually draws from sources such as EMRs, hospital information systems, drug repositories, centralized laboratory data systems, and national immunization databases [84].

^b^While covariance structure analysis can be applied to formative constructs, this requires stringent model identification conditions that are theoretically problematic or empirically inapplicable in a research model such as ours. For an endogenous construct, the identification condition requires specifying the construct’s formative indicators as endogenous variables [85].

^c^The three “EMR functional coverage” variables and the three “extended EMR use” variables are operationalized through “index” rather than “scale” measures [86]. An index variable tends to follow a Poisson-type distribution rather than a normal distribution, i.e. it is right-skewed if the mean is small. Moreover, an index comprises elements that are not expected to be highly intercorrelated, hence the inappropriateness of using Cronbach’s alpha coefficient to test its reliability [87].

^d^VIF_*i*_ = 1/(1-R_*i*_^2^) where R_*i*_^2^ is the unadjusted R^2^ obtained when component_*i*_ is regressed against all other components of the formative construct.

^e^We conducted a CFA in which an “unmeasured latent method” construct was added to the measurement model, with the measures being allowed to load on this construct as well as on their theoretical construct. The CFA allowed us to break down the variance of the measures into theoretical, random error and method components [88]. The results show that 66% of the variance was explained by the five theoretical constructs, 8% by random errors, and 26% by the method construct. Moreover, this model did not fit the data any better than a second CFA model in which the latent method construct was removed.

## Abbreviations

AVE: Average Variance Extracted
CFA: Confirmatory Factor Analysis
CPOE: Computerized Physician Order Entry
CIS: Clinical Information Systems
CMB: Common Method Bias
EFA: Exploratory Factor Analysis
EHR: Electronic Health Records
EMR: Electronic Medical Records
IS: Information System
IT: Information Technology
PACS: Picture Archiving and Communication System
QFGP: Quebec Federation of General Practitioners
SEM: Structural Equation Modeling
VIF: Variance Inflation Factor.
